# IRIS: Integrated Retinal Functionality in Image Sensors

**DOI:** 10.3389/fnins.2023.1241691

**Published:** 2023-09-01

**Authors:** Zihan Yin, Md Abdullah-Al Kaiser, Lamine Ousmane Camara, Mark Camarena, Maryam Parsa, Ajey Jacob, Gregory Schwartz, Akhilesh Jaiswal

**Affiliations:** ^1^Information Sciences Institute, University of Southern California, Los Angeles, CA, United States; ^2^Department of Ophthalmology, Northwestern University, Evanston, IL, United States; ^3^Electrical and Computer Engineering, George Mason University, Fairfax, VA, United States

**Keywords:** retina inspired sensor, neuromorphic sensor, image sensor, object motion sensitivity, Looming Detection, circuit design

## Abstract

Neuromorphic image sensors draw inspiration from the biological retina to implement visual computations in electronic hardware. Gain control in phototransduction and temporal differentiation at the first retinal synapse inspired the first generation of neuromorphic sensors, but processing in downstream retinal circuits, much of which has been discovered in the past decade, has not been implemented in image sensor technology. We present a technology-circuit co-design solution that implements two motion computations—object motion sensitivity and looming detection—at the retina's output that could have wide applications for vision-based decision-making in dynamic environments. Our simulations on Globalfoundries 22 nm technology node show that the proposed retina-inspired circuits can be fabricated on image sensing platforms in existing semiconductor foundries by taking advantage of the recent advances in semiconductor chip stacking technology. Integrated Retinal Functionality in Image Sensors (IRIS) technology could drive advances in machine vision applications that demand energy-efficient and low-bandwidth real-time decision-making.

## 1. Introduction

Animal eyes are extremely diverse and specialized for the environment and behavioral niche of each species (Land, 2005). Specialization is particularly robust in the retina, a part of the central nervous system containing parallel circuits for representing different visual features. In contrast, the engineered “eyes,” *i.e.*, image sensor technology used in machine vision, are highly stereotyped. Even though cameras can have different optics on the front end, the image sensor chip, which represents the electronic analog of the biological retina, is essentially a two-dimensional array of pixels, each transmitting a luminance signal at a fixed frame rate (El Gamal, 2002). A motivating hypothesis for the present work is that 3D integration technology can be leveraged to embed retina-inspired computations onto image sensors—nicknamed IRIS (Integrated Retinal Functionality in Image Sensors) cameras—to generate highly specific feature-selective computational spikes similar to their biological retinal counterparts.

Rod and cone photoreceptors form the input layer of the vertebrate retina, where they transduce light into an analog voltage signal that is then transmitted via *bipolar cells* to the inner retina. Signals diverge at this first synapse from each cone photoreceptor onto approximately 15 different bipolar cell types (Eggers and Lukasiewicz, 2011). Functional divergence and the sophistication of visual processing then increase dramatically in the inner retina where more than 60 *amacrine cell* types (Yan et al., 2020) shape the signals to implement various computations. Finally, signals from bipolar and amacrine cells are collected by over 40 types of *retinal ganglion cells* (RGCs), the output cells of the retina whose axons form the optic nerve (Goetz et al., 2022).

RGCs transmit spike trains containing information about specific visual features like object movement, direction, orientation, and color contrast (Sernagor et al., 2001). Each RGC type provides a full representation of visual space. Thus, while the input layer of the retina is analogous to an analog pixel array (albeit one with built-in filtering and gain control), once the photoreceptor signals have been processed by the dozens of cell types comprising retinal circuits, the output representation is very different, representing specific visual features. Binary RGC spike trains convey information about more than 40 different visual features to the brain. Each point in the visual space is represented in parallel in all the feature-selective RGC outputs.

Solid-state circuits that mimic detailed electrochemical behavior of retinal cells and associated circuits date back to the advent of neuromorphic computing in the 1980s (Mead and Mahowald, 1988). More recent works have shown how complex interacting retinal cells can lead to known retinal circuit functionalities, including the detection of differential motion (Tseng and Parker, 2012). Note these works aimed to mimic detailed electrochemical processes of retinal cells using analog electronic circuits and were not focused on image sensor or camera technology. Interestingly, efforts to bring biologically-inspired functionality to electronic image sensors gained widespread attention with the advent of neuromorphic sensors (reviewed in Liu and Delbruck, 2010; Zhu et al., 2020). Two related aspects of visual computation, which were already well characterized in retinal neurobiology by the late 1980s, have dominated the field of neuromorphic vision sensors. The first idea was to mimic luminance adaptation, the computation used by the retina to adjust the dynamic range of its biological components to that of the visual scene. Humans use vision over 10 orders of magnitude of luminance (Rodieck, 1998), and even single natural images vary in brightness by more than a factor of 10^5^ (Frazor and Geisler, 2006). Linear photodiodes and digitization to 8 or even 12 bits represent these high dynamic ranges. High dynamic range (HDR) cameras use multiple exposures to reconstruct an image, trading bit depth for frame rate (Schanz et al., 2000), while logarithmic detectors use range compression to avoid saturation (Bae et al., 2016). The second aspect of retinal computation to take hold in neuromorphic image sensors is change detection, the propensity of retinal neurons to adapt to the mean luminance over time and only transmit information about its change. Event-based cameras, or Dynamic Vision Sensors (DVS), implement temporal differentiation at each pixel and asynchronously transmit binary ‘spike' events when the luminance change exceeds a threshold. The asynchronous transmission of DVS cameras has critical advantages for high-speed operation since it is not limited by frame rate and for efficiency, since pixels that do not change do not transmit data (reviewed in Etienne-Cummings and Van der Spiegel, 1996; Liao et al., 2021).

This work presents a new class of neuromorphic sensors called *Integrated Retinal Functionality in Image Sensors (IRIS)*. By leveraging recent advances in understanding inner retinal circuits, IRIS technology goes beyond luminance adaptation and change detection features mostly confined to phototransduction and the first retinal synapse to implement computations that occur in the circuits of the inner retina, mimicking the feature-selective spike trains of RGCs. Here we present IRIS circuits implementing two retinal motion computations: *Object Motion Sensitivity* (OMS) and *Looming Detection* (LD). The present work aims not to implement the detailed electrochemical dynamics of retinal cell types but to functionally mimic the computational behavior of retinal circuits on state-of-the-art image-sensing platforms.

OMS is a computation that enables the visual system to discriminate the motion of objects in the world (object motion) from motion due to one's eye, head, and body movements (self-motion) (reviewed in Baccus et al., 2008). A subset of RGCs responds to either local motion in the receptive field ‘center' or differential motion of the receptive field ‘center' and ‘surround' regions but remains silent for global motion (Yu et al., 2019). OMS RGCs are thought to be important in detecting movements of predators and prey amidst a background of self-motion (Schwartz, 2021). For machine vision applications, a fast sensor with built-in OMS could detect moving objects even if the camera was moving, for example, on an autonomous vehicle.

LD is a computation that likely evolved to warn animals of approaching threats, especially those from overhead (reviewed in Temizer et al., 2015). Loom-sensitive RGCs respond selectively to expanding (approaching) dark objects with much weaker responses to translational motion across the visual field (Münch et al., 2009). Experiments in flies (Card, 2012), zebrafish (Temizer et al., 2015), frogs (Ishikane et al., 2005), and mice (Yilmaz and Meister, 2013; Wang et al., 2021) have established a causal role for LD RGCs in eliciting stereotyped escape responses. In machine vision, an LD-equipped sensor could be used on an autonomous vehicle to avoid collisions by enabling fast detection of approaching objects.

We show OMS and LD circuits built on standard Complementary Metal Oxide Semiconductor (CMOS) technology based on active pixel sensors (APS) and DVS pixels. We exploit advances in semiconductor chip stacking technology and highly scaled, dense CMOS transistors to embed retina-inspired circuits in a hierarchical manner analogous to the processing layers of the biological retina. Our simulations demonstrate the prevalence of OMS and LD triggering stimuli in natural scenes from moving vehicles, and they show circuit designs that implement both the OMS and LD computations and are compatible with existing image sensor fabrication technology. This work forms the necessary foundation to build IRIS-equipped cameras for machine vision.

## 2. Materials and methods

Hardware circuit simulations were performed using the process design kit (PDK) from Globalfoundries for fully-depleted Silicon-on-insulator (FD-SOI) technology at 22nm technology node (Shilov, 2015). The 22nm FD-SOI node is well-suitable for the mixed signal circuits used in this work (Gorss and McGill, 2015). The simulations were run on industry-standard EDA (Electronic Design Automation) tools from Cadence (Cadence Newsroom, 2022).

## 3. Result

### 3.1. Algorithmic implementation of retinal computations

**Figure 1:**
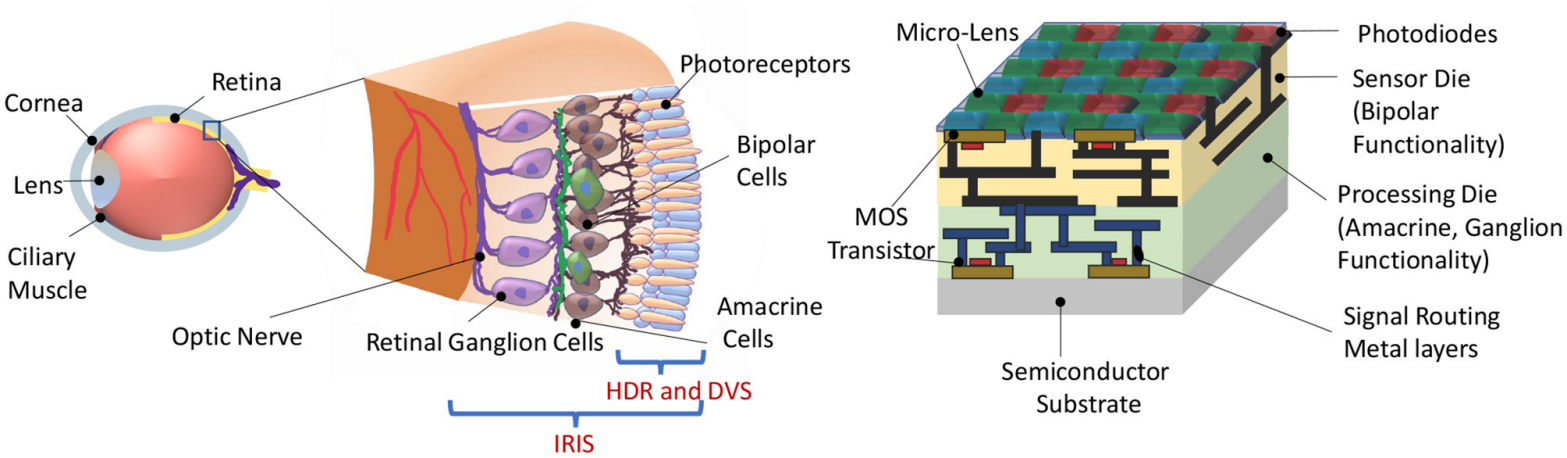
**(Left)** Representational view of the biological retina. **(Right)** Proposed IRIS camera implementing retinal computations on a back-side illuminated active pixel sensor (Okada et al., 2021) camera with Bayer pattern color filters (Catrysse and Wandell, 2005). Two retina-inspired functionalities in existing works are HDR (High Dynamic Range) and DVS (Dynamic Vision Sensor), confined to phototransduction and the first retinal synapse. In contrast, the proposed IRIS camera aims to implement computations that occur in the circuits of the inner retina, mimicking the *feature-spikes* of Retinal Ganglion Cells (RGC).

Feature selective circuits in the vertebrate retina, like OMS and LD, are built from 5 classes of neurons (Figure 1). Photoreceptors construct the input layer (like the pixels in a camera), and retinal ganglion cells (RGCs) represent the output. The computations that transform the pixel-like representation of the photoreceptors to the feature-selective representation of RGCs are carried out by the 3 interneuron classes: horizontal cells, bipolar cells, and amacrine cells. Horizontal cells are mainly involved in lateral inhibition and color processing, but they do not play a major role in OMS and LD circuits (Schwartz, 2021). Thus, we designed the components of IRIS circuits to match the functionality of bipolar, amacrine, and ganglion cells in these computations.

**Figure 2:**
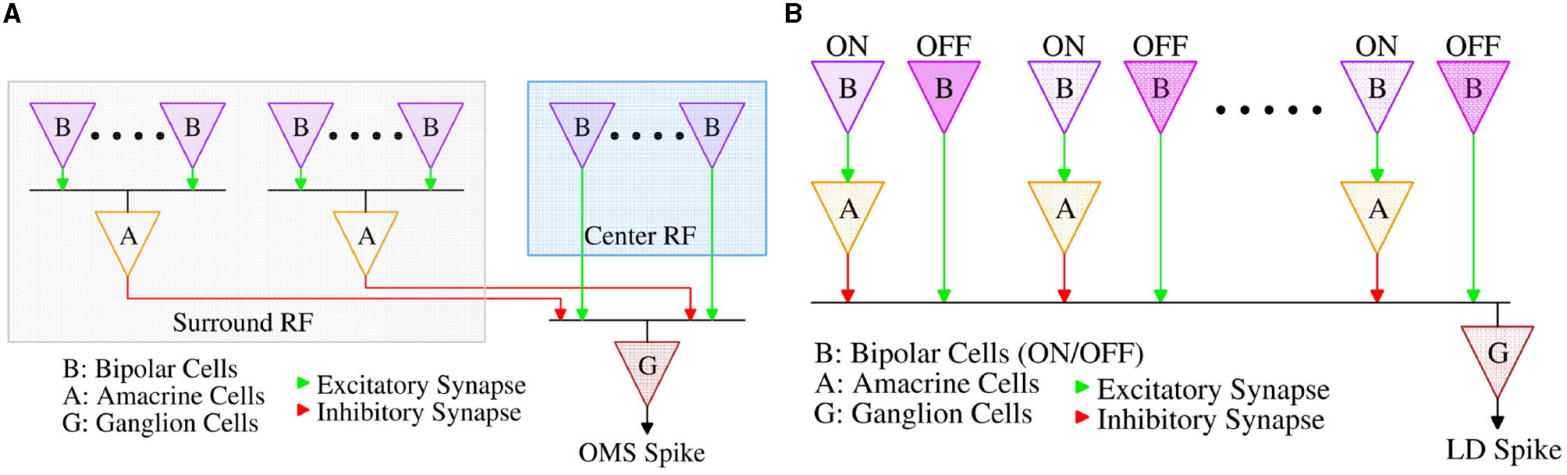
**(A)** Retinal Object Motion Sensitive circuit, and **(B)** Retinal Looming Detection circuit.

Both computations begin with bipolar cells that act like differentiators; they adapt (rapidly) to steady illumination and signal-only changes in luminance. In the biological retina, separate bipolar cells carry signals for positive (ON), and negative (OFF) changes in illumination. The OMS retinal circuit (Figure 2A) combines this functionality at the level of ON-OFF bipolar-like units; on the other hand, the LD circuit Figure 2B has separate ON and OFF bipolar sub-circuits.

Amacrine cells are the most diverse class of neurons in the retina, comprising more than 60 cell types (Yan et al., 2020). While many of the cellular and synaptic details of amacrine cells remain incompletely understood, their algorithmic role in the OMS and LD circuits has been well characterized (Baccus et al., 2008; Zhang et al., 2012), reviewed in Schwartz (2021). In the OMS circuit, amacrine cells collect the bipolar cells' contrast responses from a wide spatial extent, the receptive field “surround”, and relay this summed signal with an opposite sign to the output of bipolar cells from the receptive field “center”, implementing a spatial filter with a subtraction operation, thereby detecting differential motion between the “center” and the “surround” regions. In the LD circuit, amacrine cells also invert the sign of signals from bipolar cells but on a smaller spatial scale. OFF signals from the leading edge of a dark moving object are relayed directly by OFF bipolar cells to RGCs, while ON signals from the trailing edge of the object are relayed with the opposite signs to the RGC via intermediary amacrine cells. Thus, moving objects with both leading and trailing edges elicit opposing responses that cancel at the level of the RGC, while expanding dark objects with only leading edges elicit an RGC response. A more detailed description of the functioning of OMS and LD circuits can be found in Schwartz (2021) and Gollisch and Meister (2010).

**Figure 3:**
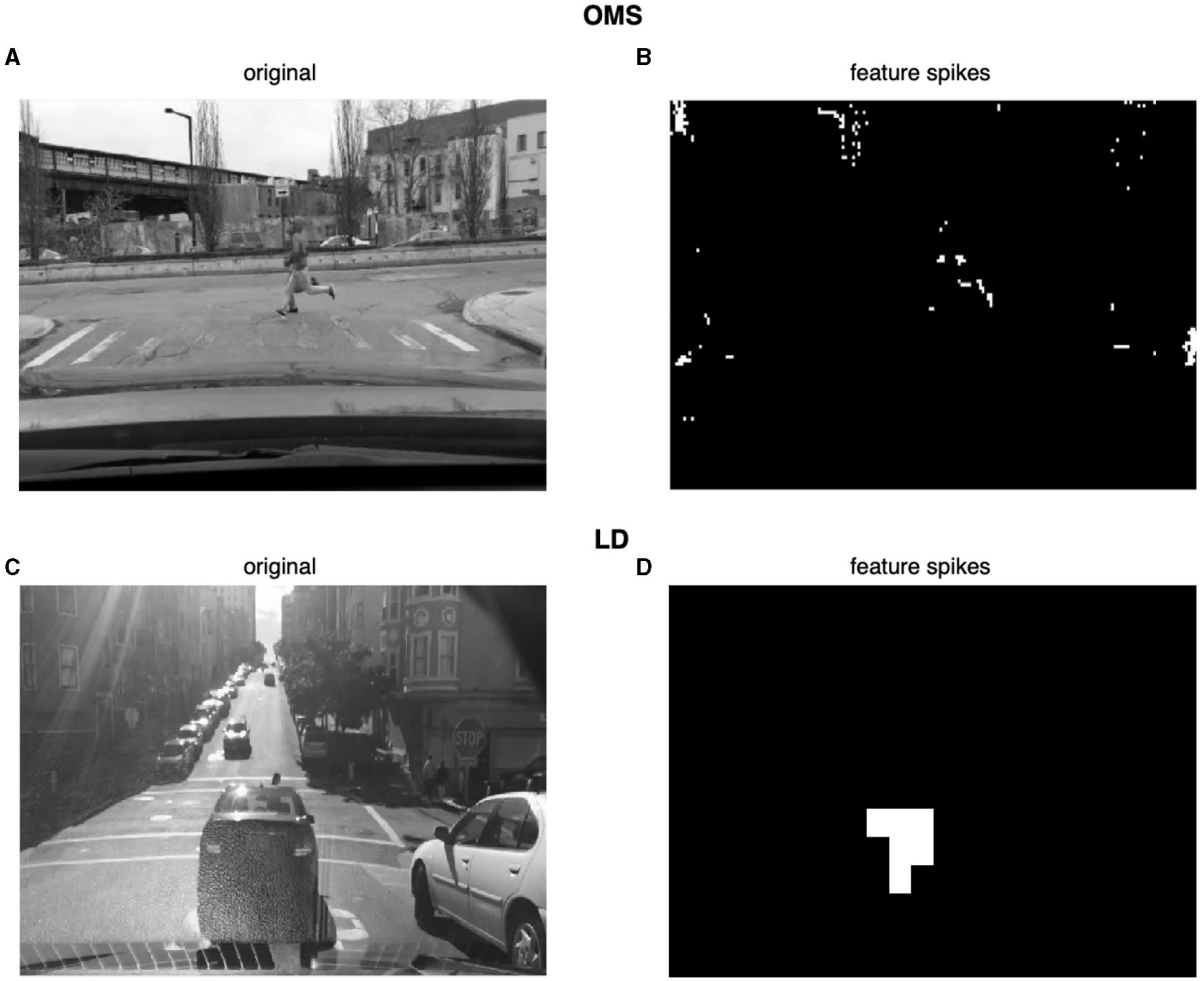
Example frames showing the software implementation of the OMS and LD algorithms. **(A, C)** are frames from the Berkeley DeepDrive database Yu et al. (2020). Frames in **(B, D)** show the corresponding sparse representation at the output of the OMS and LD circuits, respectively. White pixels indicate spike events. Spikes can be seen in frame **(B)** indicating the identification of differential motion (runners in front of a moving car) in accordance with the expected OMS behavior. Similarly, frame **(D)** shows spikes indicating a looming (approaching) car in the receptive field.

Before designing the electronics for IRIS sensor hardware, we confirmed our algorithmic understanding of the OMS and LD computations by implementing them in software and testing them on dashboard video segments from the Berkeley DeepDrive database (Chen et al., 2015). The OMS algorithm elicited simulated RGC spikes for the expected features of the videos, like runners crossing the street in front of the moving car (Figures 3A, B). Likewise, the LD circuit signaled the approach of negative contrast (dark) objects (Figures 3C, D). Based on these results, we sought to design hardware IRIS circuits using the mixed-signal design that implemented the same OMS and LD computations as our software.

### 3.2. Embedding OMS functionality in image sensors

**Figure 4:**
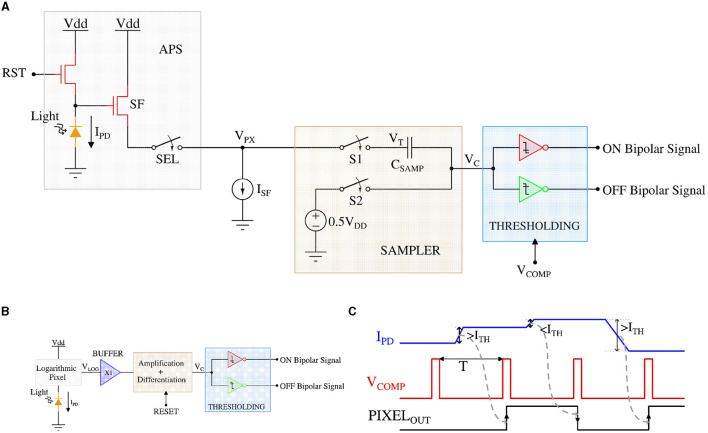
**(A)** CMOS implementation of the APS pixel circuit diagram, **(B)** DVS pixel circuit diagram, and **(C)** the timing waveform of the retinal bipolar functionality using APS and DVS pixel.

As described above, the OMS computation in the retina starts with detecting changes in the temporal contrast of input light by the bipolar cells. In other words, for OMS behavior, functionally, the bipolar cells generate an electrical signal for a change in light intensity above a certain threshold. Figures 4A, B show solid-state circuits that can mimic the bipolar cell's contrast-sensitive behavior using conventional CMOS Active Pixel Sensor (APS) (Chi et al., 2007) and Dynamic Vision Sensor (DVS) (Son et al., 2017), respectively. Note, APS pixels are of specific importance since they form the backbone of state-of-the-art camera technology (Park et al., 2021) and a wide class of computer vision applications (Voulodimos et al., 2018).

#### 3.2.1. Proposed contrast-change detection circuit

For APS-based implementation, the focal plane array is formed by a 2-dimensional array of APS pixels with the additional circuit to enable temporal light contrast-change detection. The array of such contrast-change sensitive APS pixels sample the input light intensity for each frame in parallel and compare it to the light intensity of the next frame. If the light intensity sensed by each APS pixel increases (decreases), the contrast-sensitive APS pixels would generate an ON (OFF) *bipolar-signal*.

Consider the APS-based contrast-change detection circuit of Figure 4A. For the APS pixels, the output voltage of the well-known 3 transistor pixel circuit is inversely linear proportional to the incident light intensity (Kleinfelder et al., 2001).

The SAMPLER block samples the pixel output in parallel for each frame and performs analog subtraction operation between two consecutive samples (or frames) for each pixel simultaneously. The subtraction operation starts by sampling the 3T APS pixel voltage (V_PX_) of the first frame on the top plate and a constant 0.5V_DD_ on the bottom plate of the sampling capacitor (C_SAMP_). In the next frame, the bottom plate of the sampling capacitor is left floating, whereas the top-plate samples the consecutive frame's pixel voltage. As a result, the floating bottom plate of the capacitor (node V_C_) follows the top plate of the capacitor and stores the difference voltage of the two consecutive frames offset by a constant voltage of 0.5V_DD_. Finally, the difference voltage (corresponding to the intensity or contrast change for a given pixel between two consecutive frames) on the bottom plate of the sampling capacitor is compared to a threshold using the THRESHOLDING circuit [implemented using two transistor static inverter-based comparators (Son et al., 2017)]. The THRESHOLDING circuit generates a spike through the ON (OFF) channel if the light intensity has increased (decreased) between two consecutive frames. Note, the array of contrast-sensitive APS pixels operate *synchronously* (when the V_COMP_ is HIGH) generating a *bipolar-signal* for changes in light intensity between two consecutive frames.

In the DVS-based contrast-sensitive pixel circuit (Figure 4B), a logarithmic photoreceptor transduces the incident light into a logarithmic output voltage (Pardo et al., 2015). A source follower buffer (X1) isolates the sensitive pixel node (V_LOG_) and the following difference amplifier. The difference amplifier is implemented as a capacitive feedback amplifier that can calculate the voltage gradient corresponding to incident light intensity change in an *asynchronous* manner (Lichtsteiner et al., 2006). Finally, the output voltage (V_C_) from the difference amplifier is compared in the THRESHOLDING circuit that is similar to the APS-based circuit and generates the ON/OFF *bipolar-signal*.

Figure 4C exhibits a representative timing waveform of the APS pixel-based *bipolar-signal* generation circuit. I_PD_ represents the photodetector current corresponding to the incident light, and PIXEL_OUT_ refers to the ON/OFF *bipolar-signal* from the pixel circuit. V_COMP_ enables the comparison between two consecutive frames with a period of T (depending on the video frame rate). It can be observed from the figure that when the photodetector current (corresponding light intensity) difference in both directions is higher (lower) than the threshold (I_TH_), PIXEL_OUT_ generates a high (low) signal output and that is updated according to the frame rate. Only the waveform of the APS pixel-based circuit has been shown, as most of today's commercial cameras are using the APS pixel. However, the timing waveform of the DVS pixel-based circuit is similar to the APS pixel-based circuit, except that DVS pixels generate asynchronous spikes and are not based on the timing of signal V_COMP_.

**Figure 5:**
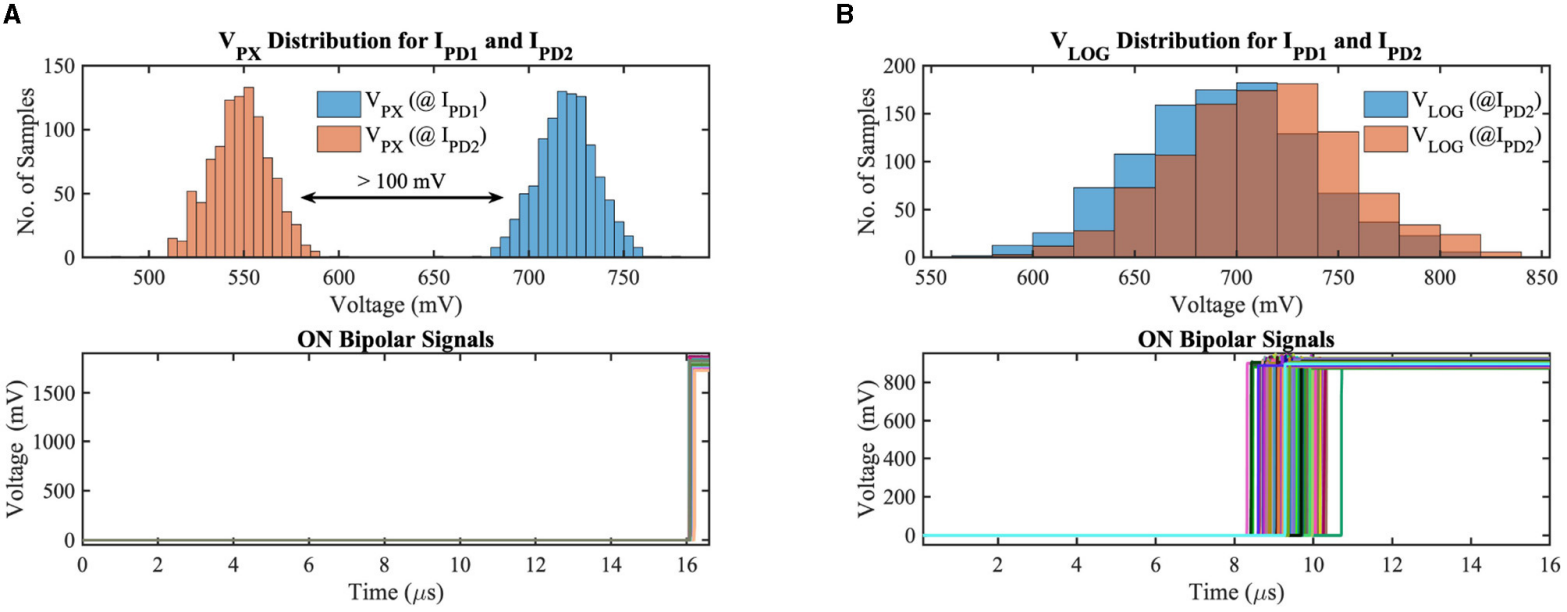
**(A)** Photoreceptor output voltage distribution for two different illuminances and ON bipolar signals in **(A)** APS-based and **(B)** DVS-based circuits considering local mismatch and global supply voltage variation on GF 22 nm FD-SOI node.

To validate our circuit's bipolar signal functionality, we have simulated the APS and DVS-based contrast change detection circuits considering the local mismatch and global supply voltage variation on the GF 22nm FD-SOI node. Figure 5 exhibits the monte-carlo simulation results for 1000 samples of our implemented circuits considering positive contrast change only. I_PD1_ andI_PD2_ represent the photodetector current (illuminance) of two consecutive frames (I_PD2_>I_PD1_) with 30% contrast sensitivity (contrast sensitivity can be defined as the ratio of minimum luminance change to absolute luminance between two consecutive frames at which the contrast-change detection circuit generates the ON/OFF bipolar signals depending on the direction of contrast change). In addition to local mismatch, we have utilized a 10 mV standard deviation to the nominal supply voltage to incorporate the supply voltage variation in our test simulations. Figure 5A shows the results for the APS-based system, where, at the top subplot, the photoreceptor voltage distribution for two different luminance levels has been demonstrated. For a co nventional 3T APS circuit, the output pixel voltage is inversely linear proportional to the incident light; hence, the mean pixel output voltage for I_PD1_ and I_PD2_ are 720 mV and 548 mV, respectively. The pixel output voltage differences (V_C_ in Figure 4A) between the two consecutive frames exceed the threshold voltage, generating ON bipolar signals for all test samples, which can be observed from the bottom subplot of Figure 5A. The photoreceptor (logarithmic) voltage distribution and ON bipolar signals for the DVS-based circuit can be observed in Figure 5B. The minimum difference (worst-case scenario among the 1,000 samples) between the photoreceptor voltages of two consecutive frames is 18.4 mV (considering 30% contrast sensitivity); however, each photoreceptor output voltage exhibits a standard deviation of 43.2 mV in our exhaustive test setup; hence, the output voltage (V_LOG_) distributions for I_PD1_ and I_PD2_ overlap with each other for different test samples. The difference voltage is amplified in the capacitive feedback amplifier stage, hence, crosses the threshold voltage, generating ON bipolar signals for all test samples that can be observed from the bottom subplot of Figure 5B. Note that the DVS-based system is asynchronous; as a result, the ON bipolar signals are generated across different time instants depending on the local variation and supply voltage.

#### 3.2.2. Proposed OMS-feature-spike generation circuit

**Figure 6:**
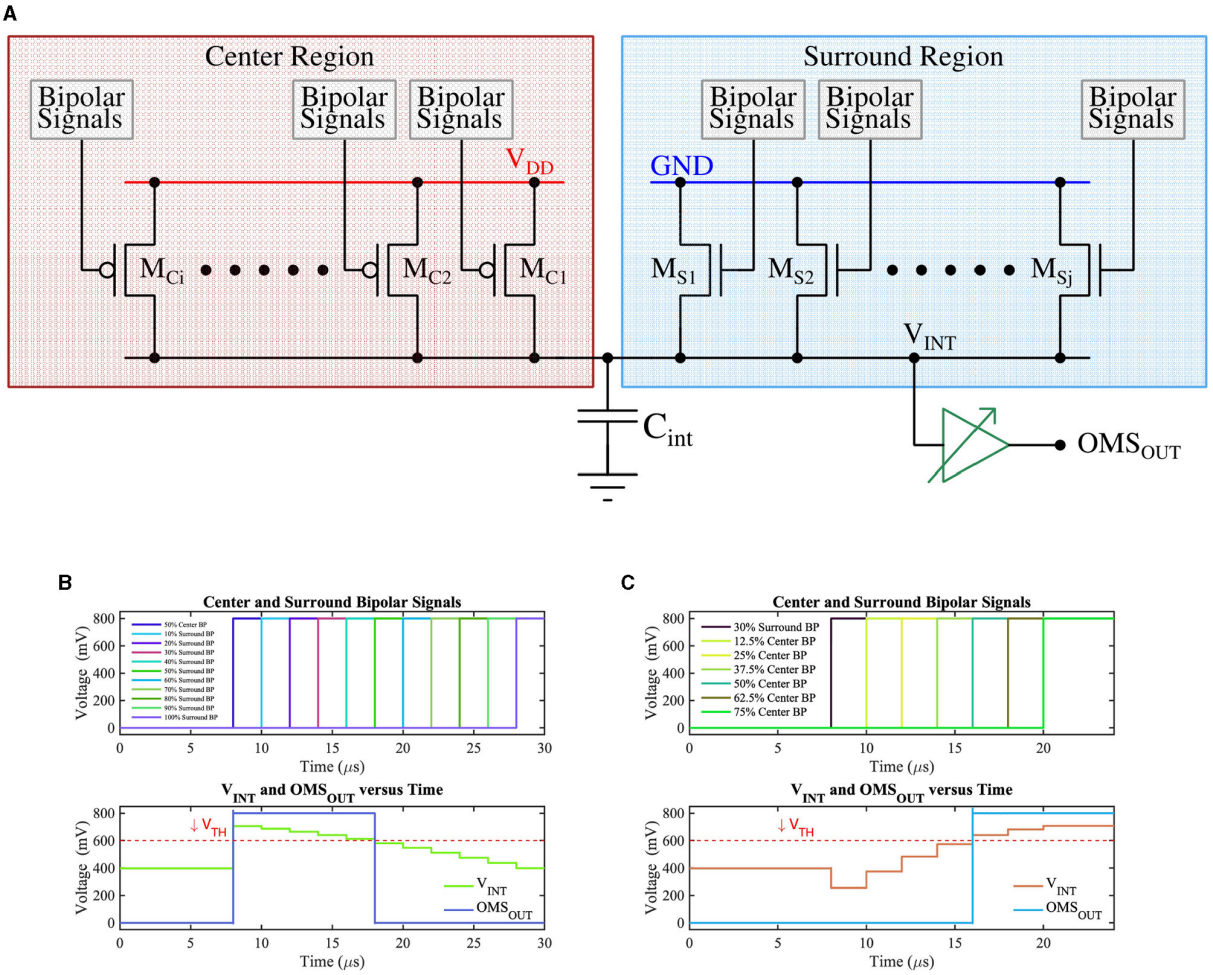
**(A)** CMOS implementation of the OMS circuit, **(B)** The voltage on node C_int_ (V_INT_) as a function of the number of ON ‘surround' transistors while keeping 50% of ‘center' transistors ON. V_TH_ represents the trip point of the buffer whose output (OMS_OUT_) represents the OMS feature spike, and **(C)** The voltage on node C_int_ (V_INT_) as a function of the number of ON ‘center' transistors while keeping 30% of ‘surround' transistors ON. V_TH_ represents the trip point of the buffer whose output (OMS_OUT_) represents the OMS feature spike.

The *bipolar-signals* generated from each pixel (either APS-based or DVS-based) are further processed by the circuit shown in Figure 6A, which implements the functionality of amacrine and ganglion cells for generation of *OMS-feature-spikes*. The circuit of Figure 6A consists of two groups of transistors, those belonging to the “center” region (transistors M_Ci_s) and those belonging to the “surround” region (transistors M_Si_s) in the receptive field. The gate of the “center” (“surround”) region transistors M_Ci_s (M_Si_s) are driven by *bipolar-signals* generated from pixels belonging to the “center” (“surround”) region. Further, the upper terminals (source) of the “center” region transistors (pFET) are connected to supply voltage V_DD_, while the upper terminals (source) of the “surround” region transistors (nFET) are connected to the ground. This ensures when a particular “center” transistor M_Ci_ receives a *bipolar-signal*, it is switched ON (active low), and *integrates* charges on capacitor C_int_. Higher the number of *bipolar-signals* generated from the center region, the higher would be the resultant voltage on the capacitor C_int_. Conversely, when a specific transistor M_Si_ receives a *bipolar-signal* from the “surround” region, it turns ON, and it attempts to drain the charge stored on the capacitor C_int_ through the ground terminal. Higher the number of *bipolar-signals* received by the “surround” transistors, the lower the resultant voltage on the capacitor C_int_. Essentially, the group of transistors M_Ci_s and M_Si_s form a voltage divider that dictates the resultant voltage (V_INT_) on C_int_. The voltage on C_int_ drives a high-skewed CMOS buffer, which generates a spike (OMS_OUT_) if the voltage on C_int_ exceeds the threshold voltage (or the trip-point) of the CMOS buffer.

In summary, when the pixels in the “center” region generate *bipolar-signals*, and at the same time, pixels in the “surround” region also generate *bipolar-signals*, it indicates that the receptive field comprising of the “center” and the “surround” region is experiencing global or background motion without any object motion. In such a case, the voltage accumulated on the capacitor C_int_ from transistors M_Ci_s in the ‘center' region is offset by the discharge effect of transistors M_Si_s in the “surround” region, and the buffer output remains low. However, if the “center” transistor M_Ci_s receive *bipolar-signals*, without significant corresponding *bipolar-signals* received by the ‘surround' transistors M_Si_s, the voltage accumulated on the capacitor C_int_ does not experience a significant discharging path through the ‘surround' pixels, resulting in higher voltage that pulls the output of the buffer high. The generated spike from the buffer, thus, represents the output *OMS-feature-spike*, indicating an object motion detected in the “center” region concerning the “surround” region.

Timing waveforms obtained by simulation of the proposed OMS circuit based on APS pixels on GF 22nm FD-SOI node are shown in Figures 6B, C. For illustration purposes, Figure 6B assumes there is no contrast change initially (before t = 8 μ s). At t = 8 μ*s*, 50% of the “center” transistors *M*_*Ci*_ have received a *bipolar-signal* and hence are ON; as a result, V_INT_ steps up from V_DD/2_ depending on the number of activations in the center region. The ‘surround' transistors *M*_*Si*_*s* are made ON such that 10% of ‘surround' transistors are ON at t = 10 μ*s*, and then the number of ON “surround” transistors increases by 10% with 2 μ*s* time-step until all the ‘surround' transistors are ON. The resulting voltage at the node *C*_*int*_ is shown in the bottom subplot of Figure 6B. As expected, node *C*_*int*_ voltage decreases as a higher percentage of “surround” transistors are switched ON. Only when sufficient ‘surround' transistors are ON, the voltage at the node *C*_*int*_ is pulled low enough (below the V_TH_) to result in a low voltage at the buffer output (OMS_OUT_).

Similarly, Figure 6C represents the test scenario where 30% of the “surround” transistors are ON (at t = 8 μ*s*), and then the number of ON “center” transistors increase by 12.5% with 2 μ*s* time-step until 75% of the “center” transistors are ON. As a result, V_INT_ increases gradually and exceeds the V_TH_, consequently, generating the OMS-feature spikes (OMS_OUT_).

We will now highlight the key design aspects of the circuit proposed in Figure 6A and its connection with the corresponding retinal OMS circuit of Figure 2A. The amacrine cells pool over a larger “surround” area as compared to the ‘center' area; this corresponds to a higher number of “surround” transistors M_Si_ compared to the ‘center' transistors M_Ci_. Pooling spikes from multiple pixels in the “surround” region is ensured in the circuit of Figure 6A, since all the “surround” pixels when activated, drive the same capacitance C_int_. Further, since the “surround” region is significantly larger than the “center” region, the signal generated from the “surround” region must be appropriately weighted by the synaptic connections to ensure proper OMS functionality. In the circuit of Figure 6A, this is ensured by designing “surround” transistors M_Si_ with lower transistor widths as compared to the center transistors M_Ci_. Finally, as shown in Figure 2A, the synaptic connections between amacrine cells from the ‘surround' region and the RGC are inhibitory; in contrast, the synaptic connections between bipolar cells in the ‘center' region and the RGC are excitatory. We ensure such inhibitory and excitatory connections by connecting the source of “center” pixels to V_DD_ and the source of ‘surround' pixels to the ground.

#### 3.2.3. Results: OMS feature-extraction circuit simulations and variation analysis

**Figure 7:**
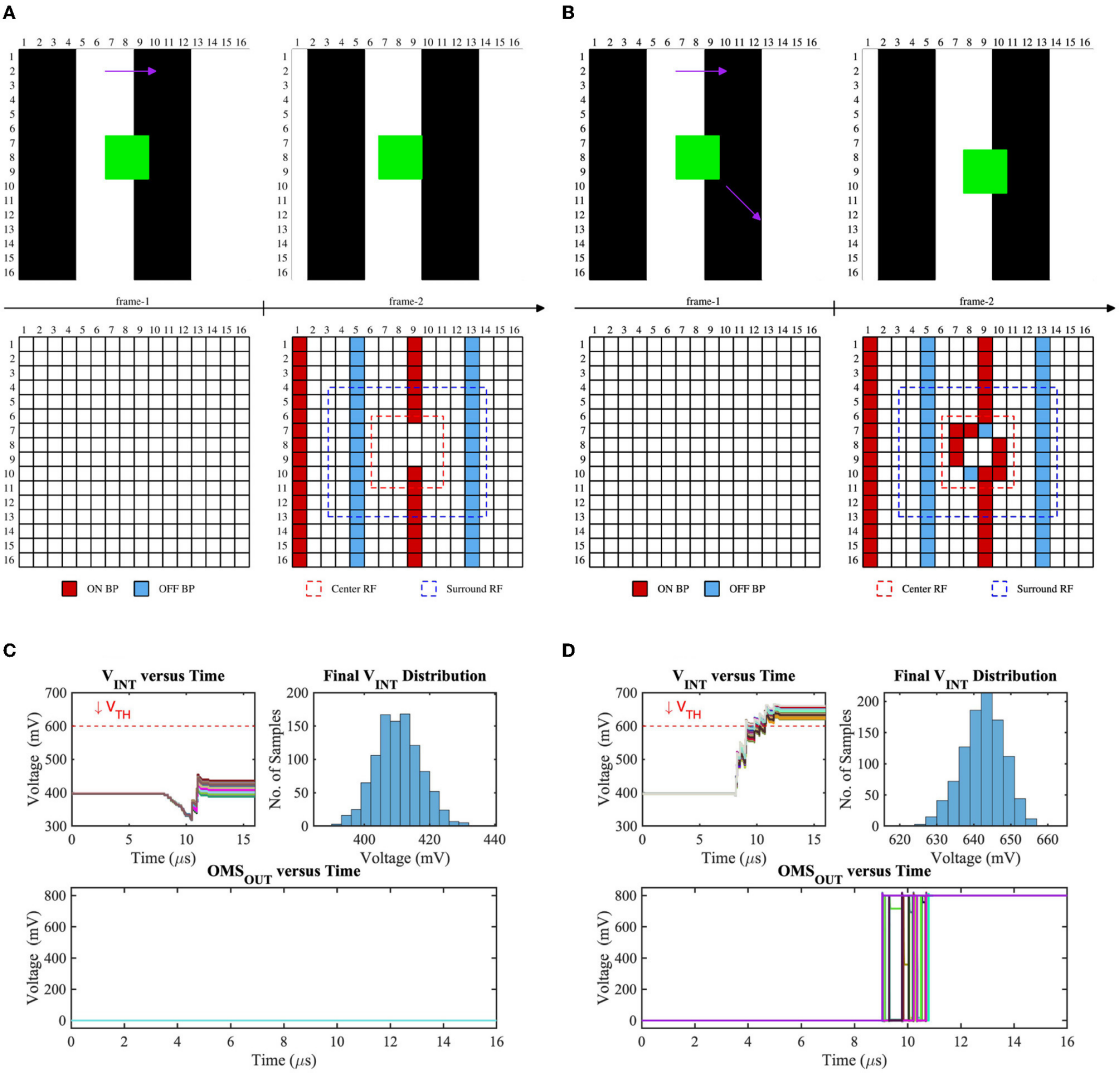
Test scenes and corresponding bipolar signals of two consecutive frames where the background is moving horizontally (global motion) while **(A)** the object is fixed, **(B)** the object is moving diagonally, V_INT_ (the voltage on the intrinsic capacitor C_int_ in Figure 6A) distribution for **(C)** test-(a), and **(D)** test-(b).

To verify the robustness of our OMS circuit's functionality, we have performed the monte-carlo simulations of 1,000 samples considering global (background) and local (object) motion. Figure 7 represents the test scenes, corresponding bipolar signals, center (red dashed) and surround (blue dashed) receptive field, voltage distribution on the C_int_ node, and OMS feature-spike (OMS_OUT_) considering global motion only (Figures 7A, C) and both global and local motion (Figures 7B, D) cases. Global motion in the visual scene will activate the bipolar signals (ON and OFF) in the center and surrounding region based on the texture of the scene and object. In contrast, the object will also generate bipolar signals based on its motion trajectory. When the object remains fixed, there will be no bipolar activations due to the object (shown in Figure 7A); in contrast, when the object is moving in a specific direction, pixels inside the center receptive field will activate bipolar signals (shown in Figure 7B). Since the surrounding region is typically larger than the center region if the number of bipolar activations in the surrounding receptive field is *sufficiently* larger than the center receptive field, the voltage on the C_int_ node will be suppressed. Hence, V_INT_ remains lower than the trip-point (V_TH_) for the fixed object scenario; as a result, no OMS spike will be generated that can be observed from Figure 7C. On the other hand, when there is a sufficient number of bipolar activations in the center receptive field due to the object motion, V_INT_ will be pulled up, consequently exceeding the threshold (V_TH_) and generating the OMS spikes (shown in Figure 7D). Note that the surrounding receptive field transistors are weighted; hence, though the absolute number of surround bipolar signals is higher than the center bipolar signals during the object motion cases, the effective pull-up strength of the circuit (Figure 6A) is higher than the pull-down network. Moreover, the variation of the timing instants of the bipolar signal activations due to local variation as well as asynchronous nature in the DVS-based system is also considered in our simulations, and that can be observed in the top-left subplots of Figures 7C, D. As seen, the final node voltages (V_INT_) are always lower (higher) than the threshold (V_TH_) during the global motion only (both global and local motion) case, ascertaining the functionality of the proposed circuit.

**Figure 8:**
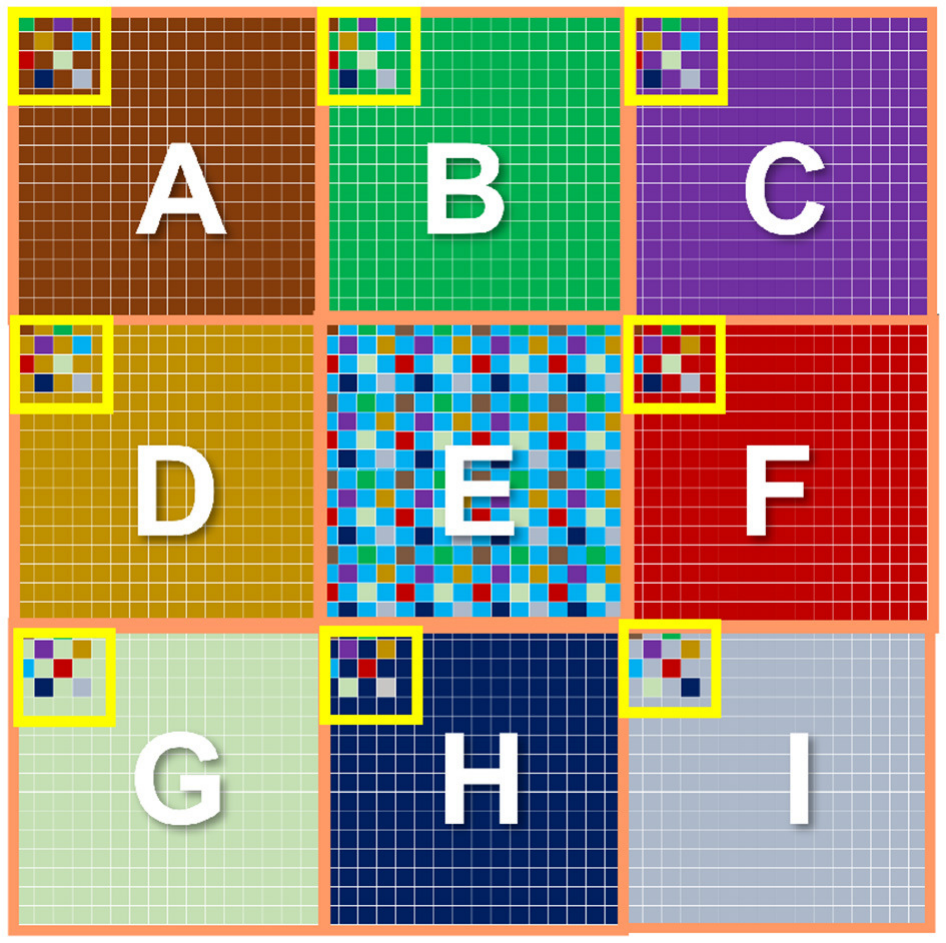
Implementation of center-surround receptive field in a 2D array of pixels.

Finally, the *center-surround* receptive field necessary for OMS functionality can be implemented in image sensors, as shown in Figure 8. Figure 8 shows a two-dimensional array of pixels. It is important to note that state-of-the-art cameras comprise millions of pixels constituting the focal plane array. We propose to divide the pixel array into multiple regions. Each region would act as a “center” region. For example, Figure 8 shows the pixel array of 9 center regions labeled *A* through *I*. Consider a specific “center” region, say region E. The “surround” region corresponding to the “center” region E is implemented as pixels interleaved in the neighboring “center” regions. Specifically, the pixels corresponding to the “center” region E are represented in blue. The “surround” pixels corresponding to the ‘center' region E are depicted as blue pixels embedded in the regions *A* through *I* except *E*. Thus, for the entire array of pixels, each “center” region will consist of most pixels constituting its own ‘center' region and fewer interleaved pixels corresponding to the ‘surround' region of neighboring “center” regions. Note, the “surround” pixel interleaving is shown explicitly for all the pixels in the “center” region *E*; in contrast, it is only shown partially for the “center” region *A* through *I* except *E*, for visual clarity. It is worth mentioning that the proposed method of Figure 8 to mimic the *center-surround* receptive field is amenable to state-of-the-art high-resolution cameras that inherently consist of numerous high-density pixels. Furthermore, the metal wires and transistors needed for routing signals between “center” and corresponding “surround” regions can be implemented using the back-end-of-line metal layers and front-end-of-line transistors from the sensor and processing die, respectively, as represented in Figure 1. Essentially, the backside illuminated CMOS sensor and the heterogeneously integrated processing chip allows transistors and photodiodes to be integrated on top of the sensor chip (which receives incident light) and another set of transistors that can be fabricated toward the bottom of the processing chip, with several layers of metals between them. Such a structure is naturally amenable to complex routing of signals as needed by *center-surround* receptive field for OMS functionality.

### 3.3. Embedding LD functionality in image sensors

#### 3.3.1. LD circuit implementation

**Figure 9:**
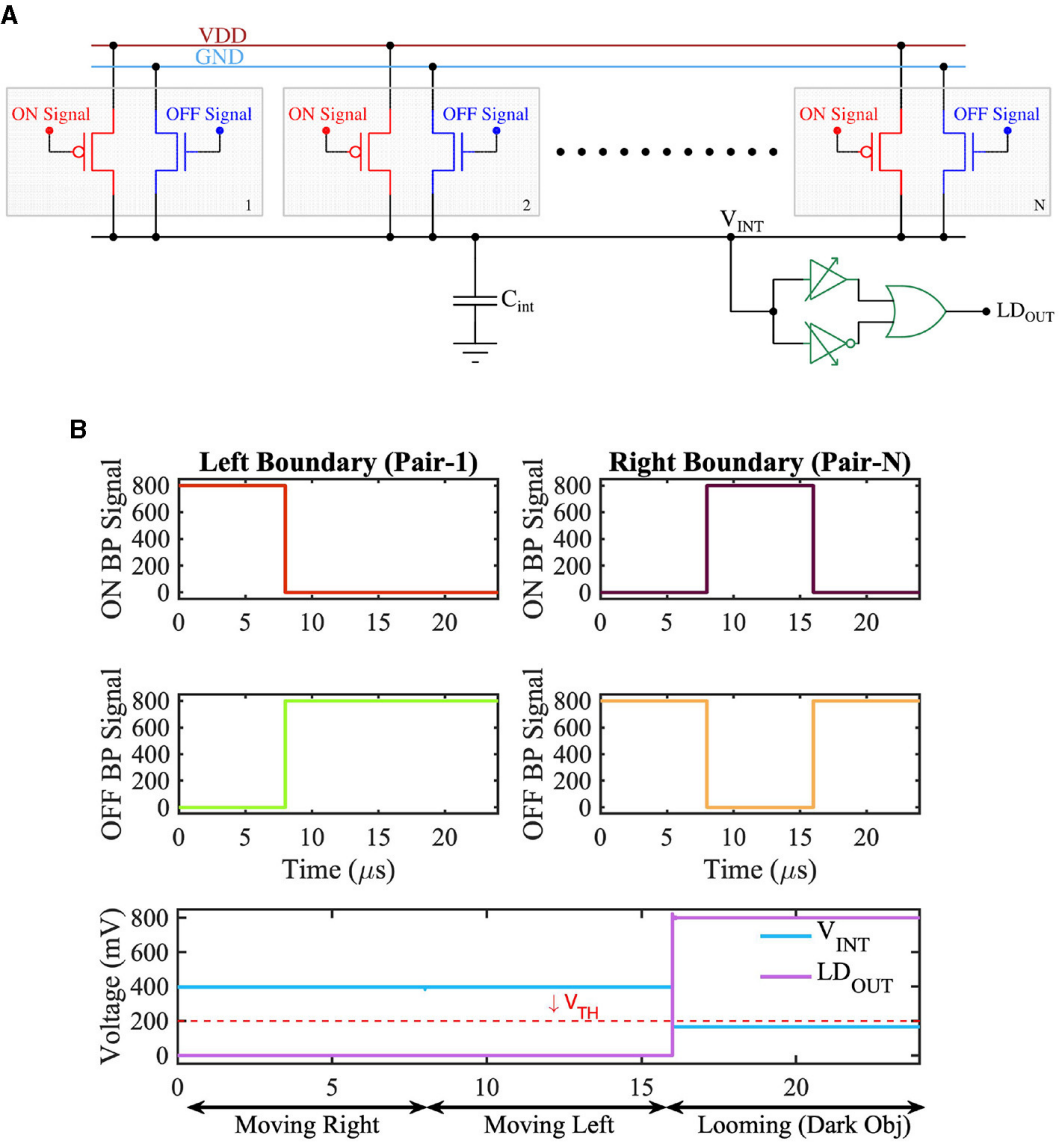
**(A)** CMOS implementation of Looming Detection Circuit diagram and **(B)** Timing waveform of the retinal Looming functionality showing the output voltage for three different scenarios.

A solid-state implementation of the retinal LD circuit from Figure 2B is shown in Figure 9A. The figure consists of multiple pairs of transistors connected to a common capacitor *C*_*int*_. Each pair consists of a transistor shown in red and another in blue. The upper terminal (source) of the red transistors (pFET) is connected to *V*_*DD*_, while the upper terminal (source) of the transistors (nFET) in blue is connected to the ground. Further, the gates of the red transistors are driven by ON *bipolar-signals*, and the gates of the blue transistors are driven by OFF *bipolar-signals*. Consider a dark object laterally moving in the receptive field. No *bipolar-signals* would be generated from those pixels in the receptive field corresponding to the dark object's internal region. This is because *bipolar-signals* are only generated in response to change in light contrast. The dark object's internal region (or body) would continuously present low light intensity and hence would not excite any *bipolar-signals*. In contrast, pixels at the object's boundary would experience contrast change as the object moves laterally. Specifically, if the dark object is moving to the right, considering Figure 9A, the pair of red and blue transistors at the left boundary of the object would experience a change in light contrast. As the dark object moves to the right, the corresponding pixel pair would experience an increase in light intensity, and an ON *bipolar-signal* would thus be generated. The ON *bipolar-signal* would activate the red transistor among the pair of transistors at the left boundary of the object. Similarly, on the right boundary of the object, an OFF *bipolar-signal* would be generated as the pixels at the right boundary would experience a decrease in light intensity as the object moves to the right. Consequently, an OFF *bipolar-signal* would be generated. The red transistor connected to the ON *bipolar-signal* at the left boundary of the object would try to pull up the voltage on the capacitor *C*_*int*_, while the blue transistor receiving the OFF *bipolar-signal* on the right would try pulling down the voltage on the capacitor. This would result in voltage on capacitor *C*_*int*_ close to *V*_*DD*_/2. The logic circuit connected to the capacitor *C*_*int*_ is designed to generate a low output when the voltage on *C*_*int*_ is within a range of *V*_*DD*_/2. The output of the logic circuit is high only when the voltage on *C*_*int*_ deviates significantly from *V*_*DD*_/2 (i.e., either is closer to *V*_*DD*_ or closer to ground). In accordance with its behavior, the logic circuit would generate a low output in response to a voltage of *V*_*DD*_/2 on node *C*_*int*_ as the object moves to the right. A similar argument holds when an object in the receptive field moves to the left, resulting in a low response from the logic circuit.

Now, consider the dark object within the receptive field is approaching (or looming). In such a case, the pair of transistors on the object's left and right boundary would simultaneously experience a decrease in light intensity, thereby generating OFF *bipolar-signals*. The blue transistors at the left and the right boundary would be activated by the OFF *bipolar-signals*, while all the other transistors would remain OFF. As such, the boundary blue transistors would pull the voltage across *C*_*int*_ low. In response to a low voltage on *C*_*int*_, the logic circuit would generate a high output voltage (or an *LD feature-spike*) indicating an approaching or looming object in the receptive field. Note, instead of a dark object, the LD circuit would also generate an *LD feature-spike* if a bright object is approaching inside the receptive field. In this case, the red transistors at the left and right boundary of the object would be active, the node voltage on *C*_*int*_ would increase closer to *V*_*DD*_, and the logic circuit would respond by generating a high output.

In accordance with the above description, Figure 9B depicts three scenarios; in the first scenario, the object is moving to the right. This leads to the generation of ON *bipolar-signals* from the lagging edge or left boundary of the object from the pair of transistors corresponding to the receptive filed at the left boundary (Pair 1 in Figure 9B). Additionally, OFF bipolar signals are generated from the leading edge or right boundary of the object from the pair of transistors corresponding to the receptive field at the right boundary (Pair N in Figure 9B). The LD circuit output stays low in this scenario. A similar argument holds when the object is moving to the left. However, for an approaching object (in the case of Figure 9B, an approaching dark object) OFF *bipolar-signals* are generated from both the left and right boundary of the object, leading to a high voltage at the output (LD_OUT_) of the LD circuit.

#### 3.3.2. Results: LD feature-extraction circuit simulations and variation analysis

**Figure 10:**
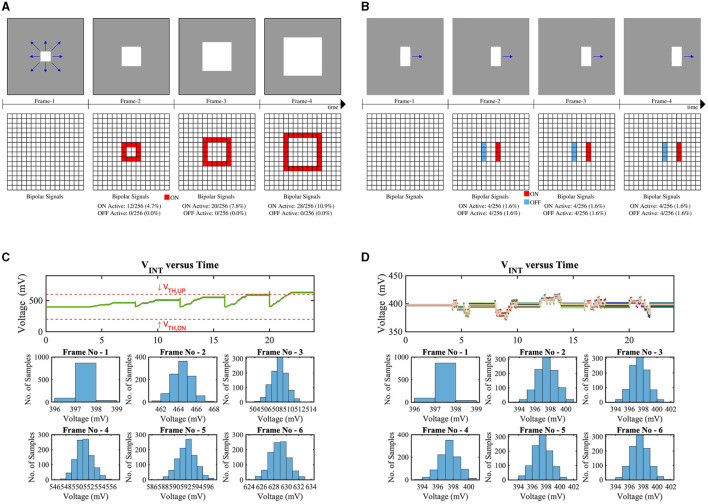
Test scenes and corresponding bipolar signals of consecutive frames where a bright square object is **(A)** approaching, **(B)** horizontally moving in the dark background, V_INT_ (the voltage on the intrinsic capacitor C_int_ in Figure 9A) distribution for **(C)** test-(a), and **(D)** test-(b).

Figure 10 exhibits the monte-carlo simulation results of 1,000 samples of our implemented LD circuit considering approaching and laterally moving objects, respectively. Figure 10A shows the consecutive frames and bipolar signal activations of an approaching bright object in the dark background. Depending on the object's distance, the number of bipolar activations will increase (the closer the object, the higher the number of bipolar activations). Due to the sufficient number of ON bipolar signal activations, the voltage V_INT_ will exceed the threshold of the skewed buffer (V_TH,UP_ ≃ 600 mV), hence, generating the LD spikes (LD_OUT_). From the bottom subplot of Figure 10C, it can be observed that during frame-6, the V_INT_ is higher than the V_TH,UP_ for all test samples. Figure 10B represents a horizontally moving object in the scene along with bipolar signal (ON/OFF) activations for consecutive frames. Due to the same number of ON bipolar activations at the leading edge and OFF bipolar activations at the lagging edge of the moving object in the receptive field, the node voltage V_INT_ remains closer to V_DD/2_ for all test samples in all the frames (shown in Figure 10D).

## 4. Discussions: future work and broader impact

IRIS sensors aim to embed retinal feature extraction behavior using retina-inspired circuits within image sensors. While the current manuscript presents two key retinal functionality embedded within image sensors—Object Motion Sensitivity and Looming Detection, similar circuit-technology design techniques can embed a rich class of retinal functionality, including color, object orientation, object shape, and more. Embedding multiple features in the IRIS camera is of special importance given the fact that individual retinal features can generate false-positive and false-negative feature spikes. By ensuring the final decision made by a computer vision algorithm (using IRIS feature-spikes) is based on multiple features as opposed to one specific feature, the overall robustness of the end application can potentially be improved. Some specific design considerations for IRIS sensors are as follows. IRIS sensors can be implemented based on underlying APS or DVS pixels. Specifically, for APS pixels to achieve high dynamic range, a coarse-grained (at pixel-array-level) or fine-grained (at individual pixel level) exposure timing control would be required (Sasaki et al., 2007; Zhang et al., 2020). For cameras with high-density APS pixels, pixels corresponding to IRIS circuits can be scattered within typical RGB (red, green, blue) pixels. At the same time, a 3D integrated chip can house the transistors and routing metal layers for implementing corresponding IRIS circuits. This would ensure the resulting IRIS camera can capture high-resolution images while simultaneously performing IRIS computations on the visual scene being captured by the camera. Further, the photodiodes associated with IRIS sensors could span a wide range of wavelengths, including visible light (Xu et al., 2005), near infra-red light (Kaufmann et al., 2011), and infra-red (Peizerat et al., 2012). IRIS cameras generate feature spikes (e.g., OMS, LD, etc.) in an asynchronous manner. In addition, depending on the number of receptive fields in the pixel array, the number of spikes will be even lower than the DVS sensor. Hence, IRIS cameras can operate similarly to DVS cameras yielding a few μs latency. For the APS version of the IRIS cameras, the frame rate will be limited by the integration time of the APS pixel array. In short, IRIS cameras can operate at a high frame rate (low latency) similar to conventional high-speed cameras. Finally, there are inherent non-linearity associated with transistor based implementation of the IRIS circuit. For example, in Figures 6B, C, the resultant voltage on the capacitor (Vint) changes the drain to source voltage of the PMOSes and NMOSes connected to the Vint node, which in turn changes the current driving strength of the transistors. This makes Vint non-linear and dependent on the number of center and surrounding pixels that are ON. The effect of such non-linearity can be mitigated through circuit-algorithm co-design for a specific target end application and is an interesting avenue for future research.

A key technology enabler for IRIS sensors is the advances in the 3D integration of semiconductor chips. 3D integration allows the integration of routing metal layers and transistor-based circuits required for implementing spatio-temporal computations directly above (or under) the pixel array, similar to the biological retinal circuit. Such 3D integrated IRIS sensors can use various 3D packaging technologies like metal-to-metal fusion bonding (Raymundo et al., 2013), TSVs (Coudrain et al., 2013), etc. Further, heterogeneous sensors operating at different wavelengths can be co-integrated to extract retina-like feature vectors over the different light spectrums. Additionally, emerging non-volatile technologies like Resistive Random Access Memories (RRAMs) (Zahoor et al., 2020), Magnetic Random Access Memories (MRAMs) (Apalkov et al., 2013), Phase Change Memories (PCM) (Lacaita and Wouters, 2008), Ferro-electric Field Effect Transistors (Fe-FET) (Lue et al., 2002), etc. can be used for IRIS circuits, for example, to implement programmable weights for ‘center' and ‘surround' regions. The circuit complexity of the IRIS cameras depends on different parameters (e.g., spike thresholds, center receptive field size, surround receptive field size, etc.), which can be optimized and reconfigured by considering a computer vision algorithm in the loop. Specifically, parameters like threshold for OMS and LD spikes are mainly a function of the target application. For example, underwater and on-land camera would have different thresholds. They also weakly depend on the specific feature like OMS or LD. We envision such parameters can be reconfigured, wherein an intelligent computer vision algorithm utilizing output from IRIS camera can provide feedback to IRIS hardware to reconfigure itself according to task at hand.

Lastly, IRIS sensors could significantly impact computer vision in general. Today's computer vision exclusively relies on light intensity-based (APS) or light change-detection-based (DVS) pixels data collected through state-of-the-art CMOS image sensors. However, in almost all cases, the appropriate context for the pixels is missing (or is extremely vague) concerning the ‘real-world events' being captured by the sensor. Thus, the onus of processing is put on intelligent machine learning algorithms to pre-process, extract appropriate context, and make intelligent decisions based on pixel data. Unfortunately, such a vision pipeline leads to (1) complex machine learning algorithms designed to cater to image/video data without appropriate context, (2) increases in the time to decision associated with machine learning algorithms requiring to process millions of pixels per frame, (3) energy-hungry and slow access to pixel data being captured and generated by the CMOS image sensor. IRIS sensors could usher in new frontiers in vision-based decision-making by generating highly specific motion and shape-based features, providing valuable context to pixels captured by the camera. The underlying algorithms processing data generated from IRIS sensors could be based on traditional deep learning models or emerging sets of spiking neural networks that could process *feature-spikes* generated from IRIS sensors. Finally, since IRIS cameras can generally use APS pixels, they can generate feature spikes and light intensity maps as computer vision algorithms require.

## 5. Conclusion

We propose a novel family of retina-inspired cameras based on recent (past decade) discoveries in retinal neuroscience, nicknamed IRIS (Integrated Retinal Functionality in Image Sensors). IRIS cameras represent the next generation of retina-inspired visual sensors that integrate the computations corresponding to both outer and inner retinal layers, as recently discovered in retinal neuroscience. Specifically, we propose embedding two key motion computations—Object Motion Sensitivity and Looming Detection—into camera-compatible hardware by leveraging 3D chip integration technology. Our proposal forms the necessary foundation to build the next generation of retinal computation-inspired cameras for machine vision applications in dynamic environments.

## Data availability statement

The raw data supporting the conclusions of this article will be made available by the authors, without undue reservation.

## Author contributions

AJai conceptualized the idea. AJai and GS designed the overall approach for IRIS sensors. ZY, MK, and MC performed circuit simulations. GS and LC implemented software code for retinal computations. AJac helped in creating a chip manufacturing roadmap for IRIS sensors. MP helped in providing a vision for algorithmic implications of IRIS sensors. All authors contributed in writing and reviewing the paper.

## References

[B1] Apalkov D. Khvalkovskiy A. Watts S. Nikitin V. Tang X. Lottis D.. (2013). Spin-transfer torque magnetic random access memory (stt-mram). ACM J. Emerg. Technol. Comput. Syst. 9, 1–35. 10.1145/2463585.2463589

[B2] Baccus S. A. Ölveczky B. P. Manu M. Meister M. (2008). A retinal circuit that computes object motion. J. Neurosci. 28, 6807–6817. 10.1523/JNEUROSCI.4206-07.2008 18596156 PMC6670970

[B3] Bae M. Choi B.-S. Jo S.-H. Lee H.-H. Choi P. Shin J.-K. (2016). A linear-logarithmic cmos image sensor with adjustable dynamic range. IEEE Sens. J. 16, 5222–5226. 10.1109/JSEN.2016.2562638

[B4] Cadence Newsroom (2022). Cadence Library Characterization Solution Accelerates Delivery and Enhances Quality of Arm Memory Products. Available online at: https://www.cadence.com/en_US/home/company/newsroom/press-releases/pr/2022/cadence-library-characterization-solution-accelerates-delivery.html (accessed 11 August, 2022).

[B5] Card G. M. (2012). Escape behaviors in insects. Curr. Opin. Neurobiol. 22, 180–186. 10.1016/j.conb.2011.12.009 22226514

[B6] Catrysse P. B. Wandell B. A. (2005). Roadmap for cmos image sensors: moore meets planck and sommerfeld. Digital Photog. 5678, 1–13. 10.1117/12.592483

[B7] Chen C. Seff A. Kornhauser A. Xiao J. (2015). “Deep driving: Learning affordance for direct perception in autonomous driving,” in 2015 IEEE International Conference on Computer Vision (ICCV) (IEEE), 2722–2730. 10.1109/ICCV.2015.312

[B8] Chi Y. M. Mallik U. Clapp M. A. Choi E. Cauwenberghs G. Etienne-Cummings R. (2007). Cmos camera with in-pixel temporal change detection and adc. IEEE J. Solid-State Circuits 42, 2187–2196. 10.1109/JSSC.2007.905295

[B9] Coudrain P. Henry D. Berthelot A. Charbonnier J. Verrun S. Franiatte R.. (2013). “3d integration of cmos image sensor with coprocessor using tsv last and micro-bumps technologies,” in 2013 IEEE 63rd Electronic Components and Technology Conference. Las Vegas: IEEE, 674–682.

[B10] Eggers E. D. Lukasiewicz P. D. (2011). Multiple pathways of inhibition shape bipolar cell responses in the retina. Vis. Neurosci. 28, 95–108. 10.1017/S0952523810000209 20932357 PMC3222954

[B11] El Gamel A. (2002). “Trends in CMOS image sensor technology and design,” in Digest. International Electron Devices Meeting. 805–808. 10.1109/IEDM.2002.1175960

[B12] Etienne-Cummings R. Van der Spiegel J. (1996). Neuromorphic vision sensors. Sens. Actuators A: Phys. 56, 19–29. 10.1016/0924-4247(96)01277-0

[B13] Frazor R. A. Geisler W. S. (2006). Local luminance and contrast in natural images. Vision Res. 46, 1585–1598. 10.1016/j.visres.2005.06.038 16403546

[B14] Goetz J. Jessen Z. F. Jacobi A. Mani A. Cooler S. Greer D.. (2022). Unified classification of mouse retinal ganglion cells using function, morphology, and gene expression. Cell Rep. 40, 111040. 10.1016/j.celrep.2022.111040 35830791 PMC9364428

[B15] Gollisch T. Meister M. (2010). Eye smarter than scientists believed: neural computations in circuits of the retina. Neuron 65, 150–164. 10.1016/j.neuron.2009.12.009 20152123 PMC3717333

[B16] Gorss J. McGill E. (2015). Globalfoundries Launches Industry's First 22nm fd-soi Technology Platform: Globalfoundries. Available online at: https://gf.com/gf-press-release/globalfoundries-launches-industrys-first-22nm-fd-soi-technology-platform/ (accessed 11 August, 2022).

[B17] Ishikane H. Gangi M. Honda S. Tachibana M. (2005). Synchronized retinal oscillations encode essential information for escape behavior in frogs. Nat. Neurosci. 8, 1087–1095. 10.1038/nn1497 15995702

[B18] Kaufmann R. Isella G. Sanchez-Amores A. Neukom S. Neels A. Neumann L.. (2011). Near infrared image sensor with integrated germanium photodiodes. J. Appl. Phys. 110, 023107. 10.1063/1.3608245

[B19] Kleinfelder S. Lim S. Liu X. El Gamal A. (2001). A 10000 frames/s cmos digital pixel sensor. IEEE J. Solid-State Circuits 36, 2049–2059. 10.1109/4.972156

[B20] Lacaita A. L. Wouters D. J. (2008). Phase-change memories. Physica Status Solidi 205, 2281–2297. 10.1002/pssa.200723561

[B21] Land M. F. (2005). The optical structures of animal eyes. Curr. Biol. 15, R319–R323. 10.1016/j.cub.2005.04.041 15893273

[B22] Liao F. Zhou F. Chai Y. (2021). Neuromorphic vision sensors: Principle, progress and perspectives. J. Semicond. 42, 013105. 10.1088/1674-4926/42/1/013105

[B23] Lichtsteiner P. Posch C. Delbruck T. (2006). “A 128 x 128 120db 30mw asynchronous vision sensor that responds to relative intensity change,” in 2006 IEEE International Solid State Circuits Conference-Digest of Technical Papers. San Francisco: IEEE, 2060-2069.

[B24] Liu S.-C. Delbruck T. (2010). Neuromorphic sensory systems. Curr. Opin. Neurobiol. 20, 288–295. 10.1016/j.conb.2010.03.007 20493680

[B25] Lue H.-T. Wu C.-J. Tseng T.-Y. (2002). Device modeling of ferroelectric memory field-effect transistor (femfet). IEEE Trans. Electron Devices 49, 1790–1798. 10.1109/TED.2002.80362612578132

[B26] Mead C. A. Mahowald M. A. (1988). A silicon model of early visual processing. Neural Netw. 1, 91–97. 10.1016/0893-6080(88)90024-X

[B27] Münch T. A. da Silveira R. A. Siegert S. Viney T. J. Awatramani G. B. Roska B. (2009). Approach sensitivity in the retina processed by a multifunctional neural circuit. Nat. Neurosci. 12, 1308–1316. 10.1038/nn.2389 19734895

[B28] Okada C. Uemura K. Hung L. Matsuura K. Moue T. Yamazaki D.. (2021). “7.6 A high-speed back-illuminated stacked CMOS image sensor with column-parallel kT/C-cancelling S&H and delta-sigma ADC,” in 2021 IEEE International Solid- State Circuits Conference (ISSCC), Vol. 64 (IEEE), 116–118. 10.1109/ISSCC42613.2021.9366024

[B29] Pardo F. Boluda J. A. Vegara F. (2015). Selective change driven vision sensor with continuous-time logarithmic photoreceptor and winner-take-all circuit for pixel selection. IEEE J. Solid-State Circ. 50, 786–798. 10.1109/JSSC.2014.2386899

[B30] Park J. Park S. Cho K. Lee T. Lee C. Kim D.. (2021). “7.9 1/2.74-inch 32Mpixel-Prototype CMOS image sensor with 0.64μm unit pixels separated by full-depth deep-trench isolation,” in 2021 IEEE International Solid- State Circuits Conference (ISSCC), Vol. 64 (IEEE), 122–124. 10.1109/ISSCC42613.2021.9365751

[B31] Peizerat A. Rostaing J.-P. Zitouni N. Baier N. Guellec F. Jalby R.. (2012). “An 88dB SNR, 30μm pixel pitch Infra-Red image sensor with a 2-step 16 bit A/D conversion,” in 2012 Symposium on VLSI Circuits (VLSIC). 128–129. 10.1109/VLSIC.2012.6243823

[B32] Raymundo F. Martin-Gonthier P. Molina R. Rolando S. Magnan P. (2013). “Exploring the 3d integration technology for cmos image sensors,” in 2013 IEEE 11th International Workshop of Electronics, Control, Measurement, Signals and their application to Mechatronics. Toulouse: IEEE, 1–5.

[B33] Rodieck R. (1998). The First Steps in Seeing. Sunderland: Sinauer.

[B34] Sasaki M. Mase M. Kawahito S. Tadokoro Y. (2007). A wide-dynamic-range cmos image sensor based on multiple short exposure-time readout with multiple-resolution column-parallel adc. IEEE Sens. J. 7, 151–158. 10.1109/JSEN.2006.888058

[B35] Schanz M. Nitta C. Bußmann A. Hosticka B. J. Wertheimer R. K. (2000). A high-dynamic-range cmos image sensor for automotive applications. IEEE J. Solid-State Circuits 35, 932–938. 10.1109/4.848200

[B36] Schwartz G. (2021). Retinal Computation. Cambridge: Academic Press.

[B37] Sernagor E. Eglen S. J. Wong R. O. (2001). Development of retinal ganglion cell structure and function. Prog. Retin. Eye Res. 20, 139–174. 10.1016/S1350-9462(00)00024-0 11173250

[B38] Shilov A. (2015). Globalfoundries Introduces 22nm fd-soi Process Technologies. Available online at: https://www.kitguru.net/components/anton-shilov/globalfoundries-introduces-22nm-fd-soi-process-technologies/ (accessed 11 August, 2022).

[B39] Son B. Suh Y. Kim S. Jung H. Kim J.-S. Shin C.. (2017). “640x48 dynamic vision sensor with a 9 um pixel and 300 meps address-event representation,” in 2017 IEEE International Solid-State Circuits Conference (ISSCC), 66–67.

[B40] Temizer I. Donovan J. C. Baier H. Semmelhack J. L. (2015). A visual pathway for looming-evoked escape in larval zebrafish. Curr. Biol. 25, 1823–1834. 10.1016/j.cub.2015.06.002 26119746

[B41] Tseng K.-C. Parker A. C. (2012). “A neuromorphic circuit that computes differential motion,” in 2012 IEEE 55th International Midwest Symposium on Circuits and Systems (MWSCAS). Boise, ID: IEEE, 89–92.27534393

[B42] Voulodimos A. Doulamis N. Doulamis A. Protopapadakis E. (2018). Deep learning for computer vision: a brief review. Comput. Intell. Neurosci. 2018, 7068349. 10.1155/2018/7068349 29487619 PMC5816885

[B43] Wang F. Li E. De L. Wu Q. Zhang Y. (2021). Off-transient alpha rgcs mediate looming triggered innate defensive response. Curr. Biol. 31:2263–2273. 10.1016/j.cub.2021.03.025 33798432

[B44] Xu C. Shen C. Wu W. Chan M. (2005). Backside-illuminated lateral pin photodiode for cmos image sensor on sos substrate. IEEE Trans. Electron Devices 52:1110–1115. 10.1109/TED.2005.848106

[B45] Yan W. Laboulaye M. A. Tran N. M. Whitney I. E. Benhar I. Sanes J. R. (2020). Mouse retinal cell atlas: Molecular identification of over sixty amacrine cell types. J. Neurosci. 40:5177–5195. 10.1523/JNEUROSCI.0471-20.2020 32457074 PMC7329304

[B46] Yilmaz M. Meister M. (2013). Rapid innate defensive responses of mice to looming visual stimuli. Curr. Biol. 23, 2011–2015. 10.1016/j.cub.2013.08.015 24120636 PMC3809337

[B47] Yu F. Chen H. Wang X. Xian W. Chen Y. Liu F.. (2020). “Bdd100k: A diverse driving dataset for heterogeneous multitask learning,” in IEEE/CVF Conference on Computer Vision and Pattern Recognition (CVPR).

[B48] Yu Y. Kurnianggoro L. Jo K.-H. (2019). Moving object detection for a moving camera based on global motion compensation and adaptive background model. Int. J. Cont. Automat. Syst. 17, 1866–1874. 10.1007/s12555-018-0234-3

[B49] Zahoor F. Azni Zulkifli T. Z. Khanday F. A. (2020). Resistive random access memory (rram): an overview of materials, switching mechanism, performance, multilevel cell (mlc) storage, modeling, and applications. Nanoscale Res. Lett. 15, 1–26. 10.1186/s11671-020-03299-9 32323059 PMC7176808

[B50] Zhang J. Newman J. P. Wang X. Thakur C. S. Rattray J. Etienne-Cummings R.. (2020). A closed-loop, all-electronic pixel-wise adaptive imaging system for high dynamic range videography. IEEE Trans. Circuits Syst. I: Regu Pap. 67, 1803–1814. 10.1109/TCSI.2020.2973396 36845010 PMC9957502

[B51] Zhang Y. Kim I.-J. Sanes J. R. Meister M. (2012). The most numerous ganglion cell type of the mouse retina is a selective feature detector. Proc. Natl. Acad. Sci. USA. 109, E2391–E2398. 10.1073/pnas.1211547109 22891316 PMC3437843

[B52] Zhu M. He T. Lee C. (2020). Technologies toward next generation human machine interfaces: From machine learning enhanced tactile sensing to neuromorphic sensory systems. Appl. Phys. Rev. 7, 031305. 10.1063/5.0016485

